# Neuronal Nicotinic Receptors in Sleep-Related Epilepsy: Studies in Integrative Biology

**DOI:** 10.5402/2012/262941

**Published:** 2012-12-09

**Authors:** Andrea Becchetti

**Affiliations:** Department of Biotechnology and Biosciences, University of Milano-Bicocca, Piazza della Scienza 2, 20126 Milan, Italy

## Abstract

Although Mendelian diseases are rare, when considered one by one, overall they constitute a significant social burden. Besides the medical aspects, they propose us one of the most general biological problems. Given the simplest physiological perturbation of an organism, that is, a single gene mutation, how do its effects percolate through the hierarchical biological levels to determine the pathogenesis? And how robust is the physiological system to this perturbation? To solve these problems, the study of genetic epilepsies caused by mutant ion channels presents special advantages, as it can exploit the full range of modern experimental methods. These allow to extend the functional analysis from single channels to whole brains. An instructive example is autosomal dominant nocturnal frontal lobe epilepsy (ADNFLE), which can be caused by mutations in neuronal nicotinic acetylcholine receptors. *In vitro*, such mutations often produce hyperfunctional receptors, at least in heterozygous condition. However, understanding how this leads to sleep-related frontal epilepsy is all but straightforward. Several available animal models are helping us to determine the effects of ADNFLE mutations on the mammalian brain. Because of the complexity of the cholinergic regulation in both developing and mature brains, several pathogenic mechanisms are possible, which also present different therapeutic implications.

## 1. Introduction

Strictly speaking, expressions like “integrative biology” or “system biology” are pleonastic, as the essence of biological processes is the interplay of many elements, even in unicellular organisms. Such locutions serve nonetheless to remind us that fully explaining physiological and pathological processes requires taking into account the organism context, which is easy to forget in a time that offers very powerful molecular methods. The pathogenesis of any disease is determined by a concourse of genetic, environmental, and developmental factors that produce their effects in a setting characterized by continuous interplay between cell groups, tissues, and organs. Understanding the conditions that predispose an individual to undergo such a course of events is thus a central problem of biology. Adopting a pathophysiological standpoint should considerably facilitate the task of gaining better integrative insight, because the experimental evidence related to pathology greatly exceeds the results available in other fields devoted to the organism biology. Therefore, although it seems unlikely that these issues will ever turn out to present general theoretical solutions, a more unified physiological comprehension of the biological individual would greatly advance our knowledge of virtually every aspect of general and applied biology, from the analysis of evolutionary constraints to the rational planning of therapy [1].

Genetic diseases are especially interesting in the present context, as they constitute the simplest biological perturbations of integrated physiological systems. Particularly amenable to experimental study are those diseases caused by mutant ion channels (*channelopathies *[2, 3]), because methods are available to investigate the effects of these mutations at all the relevant hierarchical levels. For example, in the case of a neurologic disease, whole brains can be studied by functional imaging and electroencephalography (EEG). Single cerebral nuclei and the interactions between nuclei can be studied by stimulating and recording in different sites by using single-cell or multisite recording techniques. Neural circuits can be also studied *in vitro* by applying similar methods to brain slice preparations, perhaps retaining part of the global connections, as in thalamocortical preparations. These methods can be applied down to the single cell and, if necessary, even the single protein (by applying single-channel recording methods to primary cultures or suitable expression systems). In practice, however, interpreting the results obtained by these methods in a coherent way is far from easy, for at least two reasons. First, the spectrum of necessary competences is very wide and the communication between researchers with different backgrounds is not always easy. Second, the results obtained at a given level may be difficult to interpret at another level. For instance, observing that a given mutant ion channel shows decreased function in *ex vivo* expression does not immediately suggest whether and how this features will be translated into hyper- or hypoexcitability *in vivo*. This is because this channel type could be simultaneously expressed in different brain regions, in both excitatory and inhibitory cells, and perhaps in different cell compartments (e.g., pre- and postsynaptic). As will be clear from the sections below, the result of such complexity is that the experimental data obtained at different levels of biological organization can sometimes appear contradictory. Use of different species only adds to the complexity. Nevertheless, ever more intense efforts to combine different approaches seems inescapable to maintain the hope of integrating in a sensible way the wealth of results available on the micro- and macroscopic features of the biological system.

Many cases are known of ion channel genes linked to epilepsy among both voltage-gated and ligand-gated ion channels [2]. In the case of the latter, the relationship between mutant receptors and the pathogenesis is often even less straightforward than it is for voltage-gated channels. Several mendelian epilepsies (Table 1) are known to be caused by mutant genes coding for neuronal nicotinic receptors (nAChRs) and GABA_A_ receptors (GABA_A_Rs; [3]), although, somewhat curiously, not for ionotropic glutamate receptors [4]. I here focus on ADNFLE, a sleep-related epilepsy linked to mutant nAChR receptors [2, 5]. Because of the possible relation between cholinergic and GABAergic transmission in ADNFLE and the general importance of inhibitory transmission in epilepsy [6], the main aspects of GABAergic system will be also briefly reviewed. After describing the main features of nAChRs and GABA_A_Rs, I will give a broad introduction to the physiology of sleep-waking cycle, and the main anatomical and functional aspects of the cerebral cholinergic and GABAergic systems. Next, I will describe the specific characteristics of ADNFLE, what is known about the mutations so far identified, and the currently available animal models of this disease. Finally, I will summarize the potential pathogenic mechanisms as well as the open questions.

## 2. Cys-Loop Family Receptors

**Figure 1:**
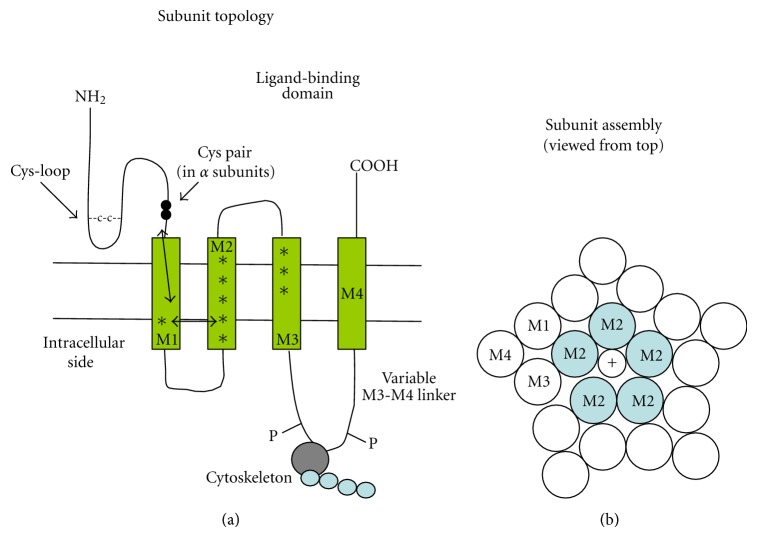
Schematic of the location of ADNFLE mutations within the nAChR subunit structure. (a) overall topology of the typical nAChR subunit; asterisks mark the location of the mutations listed in Table 2. Double arrows mark the probable transduction pathway between the ligand-binding pocket and the M2 segment, which constitutes at the same time the channel gate and the selectivity filter. The panel also shows the location of the Cys-loop and the Cys pair that defines the *α* subunits. The M3-M4 variable linker is implicated in channel interaction with the cytoskeleton and regulation by phosphorylation. (b) probable arrangement of the M1–M4 segments of the five subunits constituting the pentameric receptor. The M2 segments line the channel pore. On agonist binding, the ligand pocket partially rotates. Such conformational change is transferred to the M2 segments, whose rotation removes from the channel lumen several hydrophobic amino acid side chains. In this way, the pore diameter widens from about 0.3 nm to approximately 0.8 nm. This enlargement is accompanied by the movement of hydrophilic groups into the lumen. The overall effect is considerable increase in ion permeability. For introduction to the structure-function studies on nAChRs, see [7, 8, 12, 14, 15].

The nicotinic and the GABA_A_ receptors belong to a structural family of ion channels that also comprises the 5-hydroxytryptamine (serotonin)-gated type 3 receptors (5-HT_3_R) and the strychnine-sensitive glycine receptors. All of these are pentameric ion channels, permeable to either cations (nAChRs and 5-HT3Rs) or anions (GABA and glycine receptors). The general subunit structure is similar to the one determined for nAChRs, with a large extracellular N-terminal domain containing the ligand-binding site, four consecutive hydrophobic transmembrane domains (named M1-M4, or TM1-TM4), and a short variable extracellular C-terminus (Figure 1). Subunits can be equal or different and their stoichiometry is highly variable. Many subunits present splice variants. The name “Cys-loop” derives from the presence of a disulphide bridge which delimits 13 conserved amino acid residues forming a loop at the base of the extracellular domain (Figure 1). The Cys-loop participates in transducing the ligand-binding signal to the pore [7, 8]. In addition, the *α* subunits are defined by the presence of a Cys-Cys pair (Figure 1) in the N-terminal domain (e.g., amino acids 191 and 192 in the *Torpedo* nAChR sequence). This couple of Cys residues is necessary for ACh binding [8].

Most of what we know about Cys-loop receptors comes from studies in the nervous system and the neuromuscular junction. However, increasing evidence indicates that these receptors, particularly nAChRs [9] and GABA_A_Rs [10], are also significantly expressed in nonneuronal tissue, where their physiological roles are still matter of debate [9–11].

### 2.1. nAChRs

The nAChR [12] was discovered in the endplate postsynaptic membrane and first purified and cloned from the electroplaques of *Torpedo californica*. The history of nAChR purification, cloning, and structural characterization is an epitome of the development of concepts and methods related to ionotropic receptors [13, 14] and cannot be fully discussed here. In brief, the muscle receptor's stoichiometry in adult tissue is typically (*α*_1_)_2_ *β*_1_ *εδ*. During development, the *ε* subunit is substituted by *γ*. Therefore, in muscle receptors, two binding sites for ACh are formed by the amino acid residues located at the interfaces between *α* and *ε*/*γ* [12]. The nAChR is also widely expressed in the nervous system. To date, nine *α* (*α*2–*α*10) and three *β* (*β*2–*β*4) neuronal subunits have been cloned. The corresponding genes are named *CHRNA2-CHRNA10*, and *CHRNB2-CHRNB4* (Table 1). Subunits *α*2–*α*6 and *β*2–*β*4 can associate to form heteromeric *αβ* nAChRs, with various possible stoichiometries [12, 15]. In contrast, the *α*7–*α*9 subunits usually form homopentameric receptors. By far the most widespread is (*α*7)_5_, whereas *α*8 is restricted to avians and *α*9 and *α*10 are mainly expressed in the cochlea. However, recent evidence indicates that these subunits can also form heteromeric receptors. For example, *α*9 can associate with *α*10 [15]. Moreover, studies in expression systems show that *α*7 can associate with *β*2, even though the physiological relevance *in vivo* is unknown [16, 17]. The functional meaning of such a combinatorial diversity is unclear. In the mammalian brain, the heteropentamer *α*4*β*2 and the homopentamer (*α*7)_5_ seem to be the most common forms, at least in rodents [18], but the contribution of the other subunits is under intense investigation. For example, recruitment of *α*5 is known to modulate the properties of *α*4*β*2 receptors [19]. Moreover, a widespread expression of *α*2 has been observed in both primates [20, 21] and rodents [22]. As to *β*4, it is also widely expressed in the mammalian neocortex of squirrel monkeys [23] and adult mice [24] and is equally distributed throughout the brain in human feti and aged postmortem samples [25]. The subunit expression in different cerebral regions and nuclei is species specific; for example, *α*2 is scarcely expressed in the murine thalamus [22] but highly expressed in human thalamic samples [21]. In the peripheral nervous system, there is a prevalent contribution of *α*3 and *β*4 [26].

The overall structure of the neuronal and muscle isoforms is similar. Each subunit is constituted by a continuous polypeptide comprising approximately 500 to 600 amino acid residues. Pseudocrystalline forms of the *Torpedo *receptors [27] and X-ray crystallographic structures of the ACh-binding proteins produced by several snail species have given considerable insight into the nAChR structure [7, 8, 12]. From these studies it was inferred that these ion channels have a wide extracellular water-filled vestibule with a diameter of approximately 2 nm, formed by the long (about 200 amino acids) N-terminal extracellular domains of the five subunits. The N-terminal segment of each subunit presents a *β*-barrel structure and is followed by 4 transmembrane domains (M1–M4, or TM1–TM4). The M2 domains of the five subunits line the conduction pore and form the channel gate [28]. The variable intracellular domain between M3 and M4 is generally longer than the M1-M2 loop. It mediates channel regulation by phosphorylation [29, 30] and interacts with other cytoplasmic constituents, such as the cytoskeleton ([31]; Figure 1). The short C-terminal domains are also extracellular and present variable lengths. As is typical of ligand-gated channels as well as other allosteric proteins, at least two agonist molecules need to bind the receptor to produce significant probability that the channel is open [32]. The number of ligand-binding sites depends on subunit stoichiometry. In particular, agonists bind to a molecular crevice formed at the interface between each *α* subunit and the adjacent subunit. A good part of the binding region is contributed by the so-called C-loop in the *α* subunit, which contains the Cys-Cys pair. Other specific amino acid residues of both the involved subunits contribute to ligand binding. However, because of altered sequence, subunits *α*5, *α*10, *β*1, and *β*3 cannot participate in the agonist-binding site, although their presence regulates the nAChR properties [12]. In general, the total number of agonist molecules that bind to the receptor depends on the number of *α* subunits that contain an effective ligand-binding structure. For example, five agonist molecules can bind to (*α*7)_5_ receptors whereas only two can bind to (*α*4)_2_(*β*2)_3_ receptors ([15]). Further structural details about channel gating are given in the legend to Figure 1.

Homo- and heteropentameric nAChRs show clear functional differences. First, homomeric receptors display higher permeability to Ca^2+^. In *α*7 receptors, the permeability ratio between Ca^2+^ and Na^+^ is about 10, whereas it is approximately 2 in *α*4*β*2 receptors [33]. Moreover, in the presence of agonists, homomeric receptors desensitize more quickly and deeply than the heteromeric. Although the precise kinetics of current decay depend on subunit composition, all heteromeric nAChRs tend to display significant steady state currents in the presence of low agonist concentrations [15]. Another broad distinction between homo- and heteromeric nAChRs concerns pharmacology. The apparent affinity of *α*7 receptors for ACh and nicotine is low, with EC_50_ of about 100 to 200 *μ*M. Heteromeric receptors have instead higher affinities. For instance, the apparent EC_50_ of *α*4*β*2 nAChRs is about 30 *μ*M for ACh. This value is however somewhat misleading, as different subunit stoichiometries present different sensitivity to ligands. In particular, the concentration-response relation for ACh and nicotine of *α*4*β*2 receptors presents a high- and a low-affinity component, with EC_50_s of about 1 *μ*M and 60 *μ*M [34, 35]. These two components are, respectively, attributed to the coexistence of (*α*4)_2_(*β*2)_3_ and (*α*4)_3_(*β*2)_2_ receptors, which account for approximately 20% and 80% of the observed currents [36, 37].

### 2.2. GABA_A_Rs

The GABA receptors constitute an even wider subfamily, comprising the ionotropic GABA_A_ and the metabotropic GABA_B_ receptors. GABA_A_Rs (generally inhibited by bicuculline [38]) are heteropentamers of a variety of subunits, the main being *α*_1_–*α*_6_, *β*_1_–*β*_3_, *γ*_1_–*γ*_3_, and *δ* [39]. The corresponding genes are named, respectively, *GABRA1-A6*, *GABRB1-B3*, *GABRG1-G3*, and *GABRD* (Table 1). Additional subunits with more localized expression include *ε* (*GABRE*), *θ* (*GABRQ*), and *π* (*GABRP*). The most common subunit stoichiometry is 2*α*2*βγ*(*δ*). In addition, rho subunits *ρ*_1_–*ρ*_3_ (*GABRR1-R3*) are also known. Receptors containing a *ρ* subunit are sometimes called GABA_C_, which are mainly expressed in the retina and are insensitive to bicuculline. About 60% of the GABA_A_Rs in the brain are constituted by the *α*_1_ *β*_2_ *γ*_2_ subunits. Other common forms are *α*_1_ *β*_2/3_ *γ*_2_, *α*_2_ *β*_3_ *γ*_2_ and *α*_3_ *β*_3_ *γ*_2_ [40]. The general structure and the molecular determinants of channel assembly and interaction with other proteins are broadly similar to those observed in nAChRs [7, 8, 31]. 

GABA_A_Rs are permeable to anions. In physiological conditions, Cl^−^ is the main permeant anion, but HCO_3_^−^ also gives a significant contribution (e.g., [41]). In the adult, these channels determine the rapid postsynaptic inhibitory currents, particularly in the brain. In brainstem and spinal cord the structurally and functionally related Gly receptors tend to prevail [42]. However, certain subsets of inhibitory synapses may synthesize and corelease GABA and Gly, to generate even more complex postsynaptic responses [43, 44]. Both GABA_A_ and Gly receptors can be potentiated by a variety of extracellular agents, such as alcohols [45] and neurosteroids [46]. When the intracellular Cl^−^ concentration ([Cl^−^]_i_) reaches sufficiently high values, the reversal potential of GABA_A_ currents can make the GABAergic stimulus excitatory instead of inhibitory. This is known to occur during the development of the nervous system (see also Section 11.2). In rodents, during the second week of postnatal development, the membrane transporter NKCC (which extrudes one K^+^ and absorbs one Na^+^ and two Cl^−^) is substituted by KCC (which extrudes KCl), which is typical of the adult brain [38]. Because of the expression of different Cl^−^ transporters during brain development, in early stages the ratio [Cl^−^]_*o*_/[Cl^−^]_*i*_ is smaller than in the mature circuits. In these conditions, activating GABA_A_ receptors has depolarizing (and thus excitatory) effects. Whether a similar mechanism is also operant in the adult brain, in certain circumstances, is still matter of debate [47].

 Several idiopathic epileptic forms are caused by mutations on genes coding for GABA_A_R subunits. These are summarized in Table 1 and were recently reviewed in [48]. In brief, mutations in GABRA1 have been found to be linked to juvenile myoclonic epilepsy (JME) and childhood absence epilepsy (CAE); mutations on GABRB3 can cause CAE; mutant GABRG2 genes can be linked to febrile seizures (FS), CAE, Dravet syndrome (DS), or generalized epilepsy with febrile seizures plus (GEFS+). Finally, mutations on GABRD have been found to be linked to FS and GEFS+ [48]. 

## 3. The Sleep Stages

Sleep is a complex physiological state with distinct phases defined, in mammals and avians, by simultaneous use of EEG, electrooculography, and electromyography. The following discussion is based on references [49, 50] and summarizes the aspects relevant to the present purposes. EEG monitors the voltage fluctuations measured by electrodes applied onto the scalp. The EEG traces are determined by the coordinated activity of the thalamocortical system, which generates complex waves containing low- and high-frequency components. The electrooculogram and electromyogram register, respectively, the eye movements and the skeletal muscle tone, which further characterize specific sleep stages. A graph showing the duration and timing of sleep states is named hypnogram. During attentive wakefulness, EEG mostly comprises low-voltage (5–10 *μ*V), high-frequency (20–40 Hz) waves (*beta* rhythm). On relaxed waking, slightly ampler waves with lower frequency (about 10 Hz) tend to prevail (*alpha* rhythm). On falling asleep, alpha waves are substituted by mixed EEG waves, with frequency range similar to the beta's but somewhat higher amplitude. This phase is named *stage 1* sleep. It only lasts a few minutes and is accompanied by slow, drifting eye movements. During the following *stage 2* sleep, the EEG waves of stage 1 are punctuated by so-called *K-complex* and *sleep spindle* waves. The former are sharp, high-voltage transient waves which occur spontaneously or may be triggered by sensory stimulus. The sleep spindles are wave bursts (12 to 15 Hz) lasting 1 to 2 s, characterized by higher voltage amplitudes in the middle of the burst. This gives a spindle shape to the envelope of such waves. Stage 2 lasts about 30 min and shows no rolling eye movements. Progressively (*stage 3*), sleep spindles become more frequent and the background waves between spindles assume a higher amplitude (up to 300 *μ*V) and a lower frequency (0.5 to 3 Hz). These are called *delta* waves. During the subsequent *stage 4* sleep, delta waves predominate and spindles may disappear. Stages 3 and 4 of sleep are collectively named *slow wave sleep* (SWS), because of the prominent delta waves that reflect the synchronous activity of large populations of neurons. Stage 1 to stage 4 are collectively named non rapid eye movement (NREM) sleep, as no rapid eye movements are revealed by electrooculography. In humans, stage 4 begins approximately 1 h after sleep onset and lasts 20 to 30 min. Subsequently, the sleeper reverts to stage 3 and stage 2, from which a further state (*rapid eye movement*, or REM, sleep) can be abruptly reached. REM sleep is characterized by the beta waves typical of the waking state, but the individual is asleep and presents rapid eye movements. Moreover, the skeletal muscle tone reaches its minimum here, with diffuse peripheral paralysis. REM sleep is thought to comprise most of the oneiric activity. Its EEG waves reflect the fact that cortical neurons tend to follow an activity pattern similar to that observed in waking, with neurons not recruited in large synchronized populations. It should be however recalled that during wakefulness and REM sleep a low-voltage rhythm around 40 Hz (*gamma*) is observed. Many authors believe the gamma rhythm to reflect the temporal binding of activity in distributed neural circuits that participate in coordinated functions [51–58]. For this and other reasons, the terms synchronized and desynchronized sleep, sometimes used to refer, respectively, to SWS and REM sleep, are somewhat misleading [59]. 

In humans, several of the above NREM plus REM cycles take place during a typical nocturnal sleep, interspersed by occasional awakenings. Sleep in nonhuman mammals does not comprise exactly the same stages and is more simply described in terms of SWS and REM sleep, with durations and cycle times shorter than in humans. This is important to notice considering the widespread use of animal models of neuropathology. Moreover, voltage amplitudes can be higher when the EEG electrodes are placed directly onto the cerebral cortex of experimental animals, to perform the so-called electrocorticography [49].

## 4. Regulation of the Sleep-Waking Cycle

Moruzzi and Magoun first observed that the brainstem reticular formation maintains cortical activation and behavioral arousal during wakefulness [60]. This effect is determined by ascending stimulation of the neocortex and descending regulation of behavioral arousal and muscle tone. Neocortex activation depends on three main groups of brainstem cells: glutamatergic pontomesencephalic nuclei, cholinergic cells (laterodorsal and pedunculopontine tegmental nuclei), and noradrenergic (*locus coeruleus*) nuclei [61–63]. These nuclei exert their ascending physiological effects by following two anatomical pathways. First, they project their fibers dorsally to the thalamus, thus stimulating the nonspecific thalamocortical glutamatergic cells in the midline and intralaminar nuclei [64]. Second, they project ventrally to regulate the histaminergic cells located in the tuberomammillary hypothalamic nuclei [65] and the cholinergic and glutamatergic cells located in the basal forebrain [61]. In turn, these relay stations send diffuse projections to the neocortex and can maintain cortical activation even in the absence of the brainstem input [61]. The *locus coeruleus* also diffusely innervate the forebrain, brainstem, and spinal cord with noradrenergic fibers [61, 62].

**Figure 2:**
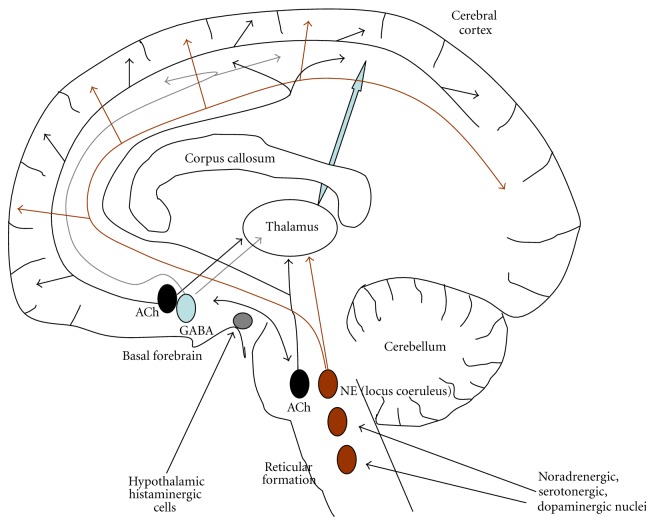
Simplified scheme of the ascending modulatory systems in the brain. The picture recapitulates the main ascending modulatory systems, with no pretension of neuroanatomical precision. Black (ACh): pontomesencephalic and basal forebrain cholinergic nuclei; light blue (GABA): GABAergic nuclei in the forebrain; brown: several noradrenergic (the main being *locus coeruleus*), dopaminergic, and serotonergic nuclei which cooperate in regulating the brain active states (see the main text); gray: hypothalamic histaminergic cells, which also regulate cortical activation. For clarity no connection pattern is shown for histaminergic, serotonergic, and dopaminergic nuclei. NE: norepinephrine.

Such an ensemble of interplaying nuclei is often referred to as *ascending reticular activating system*. A simplified scheme is given in Figure 2. Although a certain redundancy is apparent in this system, the function and the timing of activity of the different nuclei are not identical. Neurons in *locus coeruleus* maximally discharge during active waking, decrease firing during SWS, and stop firing during REM sleep [66]. In contrast, high cholinergic drive (from both pontomesencephalic and basal forebrain nuclei) is observed during both waking and REM sleep, but not during SWS [62, 67–69]. ACh release is thought to be mainly involved in neocortical activation but not behavioral arousal. Therefore, simultaneous activation of cholinergic and noradrenergic cells is necessary to sustain a waking state with both cortical activation and muscle tone. During sleep, high cholinergic activity accompanied by decreased noradrenergic tone (and lower activity of the other arousal systems) favors the transition to REM sleep, in which motor activity is largely impeded [70].

The other transmitter systems cooperate in this complex regulatory pattern. Serotonergic neurons in the brainstem *raphe *nuclei show a discharge pattern similar to the cholinergic, but the functional role is not identical, as serotonergic cells seem to be mainly implicated in less-aroused waking states [71]. They can also attenuate cortical activation by inhibiting stronger activators, such as the cholinergic cells themselves [61, 62]. Dopaminergic neurons of the *substantia nigra* and ventral *tegmentum* project to the striatum, basal forebrain, and cortex. They probably stimulate arousal in relation to the experience of positive emotions during both waking and REM sleep, while they do not seem to regulate behavioral arousal and muscle tone [72, 73]. The complexity and subtleness of the overall system are further enhanced by the reciprocal interactions of the above pathways. For example, noradrenergic fibers from the *locus coeruleus* innervate other brainstem nuclei and basal forebrain cells (including the cholinergic, [61, 62]). The arousal-controlling nuclei in brainstem, basal forebrain, and hypothalamus are also modulated by peptidergic control, particularly by hypocretin (orexin) releasing fibers [74–79]. Hypocretin neurons are located in the lateral hypothalamus; they mostly discharge during active waking and stop firing during sleep [80, 81]. Consistently, disruption of the orexin system leads to the sleep disorder narcolepsy with cataplexy in humans as well as in animal models [82–84].

The other side of the sleep-waking coin is sleep promotion. Several lines of evidence indicate that most GABAergic neurons in the basal forebrain [85–88] and preoptic area [88–90] have higher activity during SWS and cortical low-activity states (or sleep recovery). These cells are also named *cortical activation-off* and are thought to be necessary to induce sleep [91, 92]. Some of them project to the neocortex [93, 94], whereas others project locally or to the hypothalamus or brainstem [62]. A lower fraction of GABAergic cells in these regions presents instead *cortical activation-on* features [95]. In fact, basal forebrain GABAergic cells appear to be functionally heterogeneous. Some maximally discharge during wake and REM sleep, other discharge maximally during SWS, and still other progressively increase their activity during sleep, to maximally discharge during REM, in association with low electromyographic activity [96]. GABAergic cells in forebrain and preoptical area seem also to regulate hypocretin [97, 98] and histaminergic [91] cells in the hypothalamus as well as adrenergic cells of the *locus coeruleus* [91]. These cells are in their turn regulated by noradrenergic input [62]. GABAergic cells in brainstem and thalamus also appear to be active during sleep to inhibit wake-active neurons. The role of GABAergic cells in the reticular nucleus of thalamus (RT) will be described later.

## 5. The Cellular Basis of EEG in Sleep and Epilepsy

We briefly review the main cellular determinants of the EEG activity during sleep and the implication for seizure facilitation. More extensive treatment is found in [50, 59, 99–101].

### 5.1. NREM Sleep

#### 5.1.1. Mechanism of Rhythmic Burst Firing in Single Neurons

During NREM sleep, the decrease of ascending excitation hyperpolarizes thalamic and neocortical neurons (up to approximately −80 mV). Hyperpolarization favors the development of action potential bursts, particularly in thalamic cells, which are said to enter the *burst mode* of firing [102]. A more negative resting *V*_*m*_ removes inactivation from T-type (or *low-threshold*) voltage-gated Ca^2+^ channels (*I*_CaT_; [103–107]). Such a deinactivation makes *I*_CaT_ ready to activate on depolarization, thus producing a low-threshold calcium spike, whose amplitude and duration are sufficient to trigger a series of classical Na^+^-dependent action potentials [102, 108, 109]. After a burst, the cell returns to rest because of the activation of voltage- and Ca^2+^-activated K^+^ currents. Next, at negative *V*_*m*_, inactivation is again removed from *I*_CaT_. Moreover, hyperpolarization-activated cation currents (*I*_*H*_) can produce a relatively slow depolarization [110, 111], resembling the cardiac pacemaker, which can trigger another burst. This mechanism can thus give rise to rhythmic burst firing [112, 113].

#### 5.1.2. Sleep Spindles

Sleep spindles are generated by activity of the RT nucleus, which can produce spindle-like rhythms even in isolation [114, 115]. Nonetheless, the neocortex activity determines spindle synchronization and simultaneous appearance over wide regions [116]. The spindle rhythm depends on the frequency of spike bursts in GABAergic RT cells, generated as described in Section 5.1.1. As a consequence, RT neurons induce rhythmic inhibitory postsynaptic potentials (IPSPs) in thalamocortical neurons, which may lead to postinhibitory rebound spike bursts, by a mechanism analogous to the one described above. Because thalamocortical cells are excitatory, their burst activity stimulates rhythmic excitatory postsynaptic potentials (EPSPs) on neocortical cells. Occasionally, these EPSPs trigger action potentials, and more likely so during the middle portion of the EPSP train. Such a pattern gives to the overall EEG wave the typical spindle form, with higher voltage amplitude in the central phase [59]. During sleep, a large fraction of time is spent by thalamocortical neurons in spindle-related IPSPs. Therefore, these neurons are not available to transmit sensory inputs to the cerebral cortex, whereas cortico-cortical and corticothalamic dialogue may be maintained during sleep [117]. 

Although not receiving sensory stimuli, cortical neurons are actually very active during NREM sleep and overexcitation during spindles has been found to potentiate synaptic responsiveness [118, 119]. This may contribute to consolidate the memory traces acquired during wakefulness. What is more relevant in the present context, the spindle-related potentiation can lead to epileptiform activity [119]. Stimulating the thalamus and cortex with spindle-like frequencies tends to produce strong intertrain depolarizing events that can lead to paroxysmal oscillations. This phenomenon can be observed in the neocortex even in the absence of thalamus [120]. In general, seizures with spike-wave or polyspike-wave complexes tend to occur during NREM sleep. The potential relevance of these observation for ADNFLE are discussed later.

#### 5.1.3. Delta Waves

The delta waves, typical of SWS, present both a cortical and a thalamic component that can be observed independently [59]. In intact brains, however, corticothalamic volleys synchronize the thalamocortical cells through the inhibition produced by RT cells. These set the thalamocortical *V*_*m*_ at the negative value best to generate delta oscillations. Moreover, they also synchronize the dorsal thalamus. Interestingly, delta waves appear at more hyperpolarized level than spindles, in thalamocortical cells [121]. Therefore, these two rhythms are scarcely compatible. During the course of sleep, the progressive hyperpolarization of thalamocortical cells driven by the decrease in activity of cholinergic and other ascending activating fibers tends to make spindles disappear in favour of slow waves. The effect is reverted before the onset of REM sleep, in which thalamocortical cells tend to depolarize because of increased drive from brainstem [59, 101]. 

#### 5.1.4. Slow EEG Oscillation

Cortical neurons tend to exhibit cycles of spontaneous depolarizations and hyperpolarizations that produce a slow 0.7-0.8 Hz *V*_*m*_ oscillation [120, 122, 123]. This pattern also occurs during SWS and is transmitted between corticothalamic, RT, and thalamocortical neurons by following the usual pathway [123–125]. In intact brains, the sleep oscillations are thus not seen in isolation, but are grouped by the cortically generated slow wave. The combination of the slow and spindle oscillations is especially visible during light sleep [59]. The same applies to the thalamic component of delta waves. As to the intracortical component of the delta wave, the frequency band of 1–4 Hz during SWS is partly determined by the shape of the depolarizing component of the slow oscillation (0.3 to 0.4 s), that is, the K-complex. In humans, delta waves show periodic recurrence with the slow oscillation. That these two rhythms are distinct phenomena in human EEG is also evinced by the fact that delta declines from the first to the second NREM episode, whereas the slow oscillation does not [125]. In humans, stage 2 sleep shows a 0.8 Hz rhythm as well as a minor mode around 15 Hz (spindle waves).

### 5.2. Waking and REM Sleep

The major sensory and ventrolateral thalamic nuclei receive most of their brainstem innervation from the cholinergic mesopontine nuclei. In contrast, the associative and diffusely projecting thalamic nuclei mainly receive their ascending modulation from noncholinergic cells [126–128]. These pathways directly excite thalamocortical neurons and inhibit RT cells, with ensuing disinhibition of thalamocortical neurons and block of the sleep spindles. The cholinergic depolarization of thalamocortical cells presents a rapid nicotinic component and a slower sustained phase which is thought be largely caused by activation of metabotropic muscarinic ACh receptors (mAChRs; [129]). The overall effect is cortical arousal. Moreover, the effect on RT cells that leads to spindle block comprises an early short depolarization followed by a prolonged hyperpolarization, which is again probably caused by mAChR-mediated stimulation of K^+^ channels [130]. The ascending reticular arousal system also stimulates local circuit interneurons in thalamus, which may serve to enhance discriminatory tasks. A similar pattern is observed during REM sleep, in which however the brainstem drive on thalamus is supplemented by the one produced by the medulla reticular nuclei [131]. Cholinergic stimulus of the neocortex and several thalamic nuclear groups is also produced by the diffuse projections from basal forebrain cholinergic nuclei [132]. The fast oscillations of the active brain states are not limited to the cerebral cortex but also occur in thalamic neurons and are synchronized in corticothalamic networks [133]. 

The global effect is that the ascending arousal pathways depolarize the cortical and thalamic neurons, thus making them more ready to respond to sensory stimuli (during waking) or internal drives (during REM sleep). In depolarized neurons (around −65 mV), the *I*_CaT_ is largely inactive and thus insensitive to excitatory input. In this condition, thalamic neurons reside in tonic firing (or *transmission*) *mode* [113]. A sensory or internal stimulus triggers a classic action potential which is then transferred to the cortical neurons. Therefore, signals are reliably passed on to the cortex, without producing the rhythmic bursts observed in NREM sleep. This pattern of cell activity is reflected in beta and gamma EEG waves, determined by short-range synchronization of neurons involved in neocortical, thalamic, or thalamocortical circuits [53, 54, 134]. The generalized depolarization also involves inhibitory interneurons, whose increased activity shapes the dynamics of the neural circuits implicated in specific functional tasks; a process sometimes called *sculpturing inhibition* [59].

The slow oscillation described in Section 5.1.4 also groups the beta/gamma oscillations typical of waking and REM. The fast 20–50 Hz rhythms appear on the depolarizing phase of the slow oscillation because these rapid oscillations are voltage dependent [59].

## 6. The Main Features of Cholinergic Transmission in the Brain

In cholinergic terminals, ACh is synthesized from choline and acetyl-CoA by choline acetyltransferase (ChAT; [135]). Reaction occurs in the cytoplasm, from which ACh is transported into synaptic vesicles by the vesicular ACh transporter (VAChT). ChAT and VAChT are also the main immunocytochemical markers of cholinergic neurons and fibers [136–138]. They are regulated in a coordinated way, as the entire VAChT coding region is contained within the first ChAT intron, in chromosome 10 [139, 140]. Once released, ACh can activate nAChRs as well as mAChRs. The transmitter is then degraded extracellularly into choline and acetic acid, by cholinesterase enzymes. In neural tissue, the main such enzyme is acetylcholinesterase (AChE), although other cholinesterases, such as butyrylcholinesterase, exist in different tissues [135]. Choline is then taken up by the cholinergic terminals through high-affinity, Na^+^-dependent, choline transporters (ChoT, with a *K*_*m*_ for choline of about 1–5 *μ*M). These transporters have been cloned in 2000 ([141, 142], in which reference to earlier literature is found).

As discussed earlier, cholinergic fibers projecting from the mesopontine and basal forebrain nuclei profusely innervate the thalamus and cerebral cortex. ACh release cooperates with noradrenergic, histaminergic, and serotonergic transmission to increase the neocortical tone, sustain the awake state, and modulate synaptic plasticity [61, 143, 144]. ACh is also implicated in regulating the transitions between states with different level of vigilance [145, 146], included the switch between NREM and REM phases of sleep [59, 147].

The mechanisms by which ACh brings about its functions are rather intricate. Early work has tended to highlight the contribution of metabotropic mAChRs. In fact, the observed decrease in thalamic and neocortical neurons input resistance during wakefulness and REM sleep was originally attributed to a muscarinic-dependent K^+^ channel inhibition [50, 144, 148]. However, in the last two decades it has been recognized that nAChRs also exert prominent regulatory roles in the brain (e.g., [146, 149–153]). The mechanisms are only partially understood, for several reasons. First, the full physiological meaning of the combinatorial complexity of nAChR subunits is unclear. Second, nAChRs are expressed at presynaptic, postsynaptic, and nonsynaptic locations [15, 154]. In classical cholinergic synapses, ACh reaches high concentrations (around 1 mM) in the synaptic cleft and is then degraded within milliseconds by AChE. This produces a strong and brief activation of postsynaptic nAChRs. Alternatively, diffuse release by varicose cholinergic fibers can sustain low tonic ACh levels in the cerebrospinal fluid. This pattern of release determines steady state regulation of ACh receptors and is thought to be particularly important in modulating presynaptic nAChRs that are known to control glutamate and GABA release in the cerebral cortex and thalamus. Such a tonic effect is possible because the acetylcholinesterase distribution in the brain does not precisely match that of ACh receptors. Hence, stable ACh concentrations up to approximately 1 *μ*M (but normally lower) can be detected in the extracellular compartment, even in the absence of cholinesterase inhibitors [15, 154, 155]. The balance of these phasic and tonic effects is uncertain because the ratio between the effects of synaptic and paracrine transmission is difficult to assess precisely, partly because of the difficulty of counting precisely the *bona fide* synaptic contacts [156]. Nonetheless, in the rat, macaque, and murine neocortices conspicuous cholinergic varicose fibers have been observed, with rare synaptic specializations [157–159]. A further complicating element is ACh receptor expression in glial cells [160]. Because very little is known about the contribution of this aspect to neocortical synaptic transmission, this issue will not be further discussed here. Finally, intrinsic cholinergic cells have been also observed in the neocortex of rodents. The precise function of these cells is unknown, but they are thought to be implicated in the regulation of pyramidal neuron excitability [161]. Differences have been observed between rats and mice in the timing of expression of these intrinsic neocortical cholinergic cells, which is slower in mice [162, 163]. Hence, the maturation process of the nAChR-dependent control of principal cells in the neocortex may differ across mammals.

### 6.1. Physiological Roles of Heteromeric nAChRs in Neocortex and Thalamus

Heteromeric nAChRs are thought to give a major contribution to the steady state control of neocortical excitability, as they display high sensitivity to the agonists and slow desensitization. Consistent with this notion, the large majority of mutations known to be linked to ADNFLE and related pathologies map on genes coding for non-*α*7 nAChR subunits [5, 21, 164, 165]. Hence, we summarize what is known about the physiology of heteromeric nAChRs in the frontal cortex and thalamus. Although a wide literature is available about nAChR subunit expression and pharmacology, which has been partly reviewed above, relatively scarce detailed electrophysiological evidence is available on brain slices of neocortex or thalamus. On this evidence, we focus the rest of the present section.

The nAChRs are implicated in both excitatory and inhibitory transmission in neocortex and thalamus. In both rats and mice, they control glutamate release from thalamocortical fibers in the frontal cortex [166–169], which accounts for the ACh role in arousal. Moreover, evidence is also available about nAChR expression in some populations of pyramidal cells, so that direct nicotinic regulation of principal cell excitability can also take place. In fact, somatic nicotinic currents have been measured in pyramidal neurons from rat neocortical layers II/III [170], layer V [171], and layer VI [172]. The evidence in mice is less univocal [173–175], although the overall ACh effect in layer V of the prefrontal cortex seems excitatory [175]. This is consistent with the global arousing role of ACh, as cholinergic transmission in the deep layers of the prefrontal cortex can produce indirect effects on the general cortical tone. In fact, the prefrontal cortex is the only neocortical region that projects back to subcortical regions such as the basal cholinergic nuclei and the monoaminergic brainstem nuclei [176, 177]. Because layer V constitutes the main subcortical output of neocortex, it also regulates cerebral regions implicated in motor control. Therefore it may have a special role in facilitating the hypermotor activity often observed in ADNFLE (see Section 8). Moreover layer V is thought to have a special role in seizure initiation and spread [178].

Current evidence suggests that the broad features of the nicotinic regulation of GABA release are similar across mammals. Expression of nAChRs on the soma of different interneuronal populations in the cerebral cortex is established in rats [179–181], humans [182], and mice [159, 174]. In general, nAChR activation tends to stimulate GABA release in different layers. Heteromeric nAChRs also exert presynaptic control of GABA release onto pyramidal cells [159, 183]. Moreover, indirect evidence suggests that nAChRs also regulate reciprocal interneuron inhibition, at least in layer V [159]. 

The thalamus is also a major target of ascending cholinergic innervation and it was shown that presynaptic heteromeric nAChRs stimulate GABA release in thalamic nuclei in avians [184, 185] and mouse [186], whereas scarce postsynaptic effect was observed on principal cells. The glutamatergic EPSCs measured in pyramidal cells of the lateral geniculate nucleus in chick are also stimulated by heteromeric nAChRs [185]. The contribution of different subunits [185, 187] as well as the balance between nicotinic and muscarinic effects are under investigation [188]. The general pattern, at least as far as the ventral lateral geniculate nucleus is concerned, seems broadly similar to what is observed in the frontal cortex. Nicotinic receptors stimulate both excitatory and inhibitory transmission, but the overall effect is probably excitatory [188]. Activation of GABAergic transmission is nonetheless fundamental to shape and regulate the excitatory response [186]. 

## 7. GABAergic System: The Fundamentals

GABAergic cells constitute a significant fraction of the brain neurons. Most of them are inhibitory interneurons, which account for 15–25% of the total neurons [189, 190], although the precise distribution in different regions and across different layers may vary [191]. In the cerebral cortex, local GABAergic interneurons tend to prevail, but it should be kept in mind that long-range intra- and interhemispheric GABAergic projections are also present in the neocortex of cats [192–195], rodents [193, 196–200], and primates [201]. These long-range GABAergic fibers probably exert global synchronizing functions that are potentially very important in epileptogenesis [202]. Moreover, specialized GABAergic nuclei are present in the brainstem. These, along with the thalamic RT nucleus, carry out important modulatory functions over the thalamocortical system, particularly during the the sleep-wake cycle, as has been discussed earlier [59, 61].

In GABAergic terminals, GABA is synthesized from L-glutamic acid by glutamic acid decarboxylase (GAD). Differently from the case of other neurotransmitters, two isoforms are known for this enzyme, GAD65 and GAD67, encoded by independently regulated genes. GAD65 is though to be more involved in synthesizing GABA in synaptic terminals, whereas GAD67 seems mainly responsible for synthesizing cytoplasmic GABA [203]. These enzymes are often used as GABAergic markers [204]. Synaptic vesicles are loaded with GABA by vesicular GABA transporters (VGAT); the first of which was cloned in 1997 [205, 206]. VGAT is molecularly distinct from the other neurotransmitter transporters. Being electrogenic, the transport mechanism is very sensitive to *V*_*m*_. VGAT can also transport glycine (see [206] and references therein) and is expressed in glycinergic cells [207]. Hence, it is also named vesicular inhibitory amino acid transporter (VIAAT; [206]). However, current evidence suggests that other still unidentified inhibitory amino acid transporters exist in the CNS [135, 206]. After GABA is released into the extracellular space, its action is mainly terminated by uptake into glia and neurons. At least four genes coding for GABA transporters (GATs) are known and at least three GAT proteins are expressed by glial cells and neurons (not necessarily GABAergic), along with a betaine transporter that is also able to reabsorb GABA [135]. Therefore GAT proteins are not used to label specific cell types. The reasons for this diversity are unclear. One reason may be related to transporter-mediated nonvesicular GABA release. Originally observed in retinal horizontal cells [208, 209], it was next identified in central neurons and astrocytes [210, 211]. In brief, because many neurotransmitter transporters are electrogenic, changes in *V*_*m*_ accompanied by appropriate extra- to intracellular ratios of the transported ions and transmitters may lead to the transporter extruding the neurotransmitter instead of absorbing it. The specific conditions under which this process occurs depend on the stoichiometry of the transporter. A full discussion is beyond the scope of the present paper and we refer the reader to several excellent reviews [212–215]. These and other evidence indicate that GABA is also synthesized and released by astrocytes and that GABA receptors are expressed in glial cells [216], although a full view of the physiological meaning of these observations is not available.

## 8. Epilepsy and nAChRs: Autosomal Dominant Nocturnal Frontal Lobe Epilepsy

ADNFLE [217, 218] is the first idiopathic epilepsy for which a monogenic cause was demonstrated [5]. ADNFLE is a partial epilepsy characterized by clusters of hyperkinetic seizures, mostly occurring during stage 2 of sleep [217]. Functional imaging studies show that attacks arise in specific foci in one of the frontal lobes, but the location of these foci is variable. The mean duration of seizures is around 30 s. They usually begin in childhood and are sometimes misdiagnosed as nightmares or other parasomnias. No clear difference between males and females is observed. Sudden arousals are also typical of ADNFLE [218–222]. Cognitive and psychological alterations may accompany the epileptic phenotype and are probably more widespread than previously thought [223]. The clinical features of ADNFLE are very similar to those presented by the more common sporadic cases of nontraumatic nocturnal frontal lobe epilepsy [222, 224]. Therefore understanding the pathogenesis of ADNFLE should provide general information about the origin of sleep-related frontal epilepsy.

About 10–15% of the ADNFLE families characterized until now bear mutations on genes coding for nAChR subunits (Table 2). Until now, four ADNFLE-linked mutations have been identified on CHRNA4 (coding for the *α*4 subunit). All of these mutations are located on the M2 transmembrane domain [5, 225–228]. Five ADNFLE mutations have been found on CHRNB2 (coding for *β*2). Two of them are located on M2 [164, 165, 229], and three on M3 [230, 231]. Another mutation, linked to a related epileptic form, was identified on the M1 domain of CHRNA2 (coding for *α*2; [21]). Although the implication of CHRNA2 in ADNFLE seems very rare [232, 233], the above observation is interesting for comparison with the ADNFLE mutations and in that it suggests that *α*2 may also have significant roles in neocortical excitability. Figure 1 summarizes the approximate location of the different mutations. Penetrance can be as high as 80%, and a broad range of onset time and severity has been observed even among members of the same family [234]. Considering the number of independent mutations on nicotinic subunits identified in families or individuals with affine epileptic forms, the aetiologic role of the cholinergic system in ADNFLE seems well established. Moreover, mutations on nAChR subunits have also been observed in sporadic NFLE forms (e.g., [235, 236]), which reinforces the notion that nAChRs are crucial regulators of excitability, particularly during sleep. However, the genes responsible for the majority of ADNFLE forms remain to be determined and evidence suggests the existence of at least two supplementary loci [237, 238]. By now, the only ADNFLE-linked mutations identified outside the nicotinic subunit genes were found on the promoter of the corticotropin releasing hormone (CRH) gene [238]. Although the physiological meaning of this observation is presently unclear, it is interesting to notice that several lines of evidence indicate that CRH modulates the cholinergic [239, 240] and noradrenergic pathways [241]. More details about the different ADNFLE mutations are given in Table 2.

The frequent involvement of *α*4 and *β*2 in ADNFLE is particularly suggestive of the importance of heteromeric nAChRs in regulating the sleep-waking cycle. The *α*4*β*2 receptors are very widespread in the mammalian brain, and *β*2 is strongly upregulated during wakefulness and sleep deprivation [242]. Moreover, mice lacking *β*2 present longer REM phases and a less frequent interruption of NREM sleep by microarousal events [243]. The symptoms of ADNFLE and the experimental evidence obtained in patients by studying with positron emission tomography the nAChR density throughout the brain [244] indicate that epileptogenesis may depend on hyperactivity of the cholinergic system that controls cortical arousal. However, many interpretation issues arise. First, one must explain why nAChR subunits widely expressed throughout the brain induce focal attacks in the frontal lobe. Second, it must be explained why seizures tend to occur during stage 2 sleep and are accompanied by frequent sudden arousals, which points to altered thalamocortical control. Third, the ictal manifestations can be partly considered as dishinibition of subcortical activity, which manifests itself as automatic motor and limbic activity [222]. Therefore, one must determine the connection between neocortex hyperexcitability and the specific subcortical effects. Moreover, as will be illustrated in the next section, the functional properties of different mutant receptors linked to ADNFLE are not identical. Hence, it is necessary to understand whether such molecular heterogeneity may partly explain the variability of cognitive and psychological symptoms observed by ADNFLE patients [222, 223].

## 9. *Ex Vivo* Expression of Mutant Subunits Linked to ADNFLE

The functional properties of ADNFLE mutations have been studied in *Xenopus laevis* oocytes and mammalian cell lines (mainly human embryonic kidney cells). By expressing the appropriate subunit combinations, the wild type (WT), mutant and heterozygous conditions were reproduced *in vitro*. These studies, summarized in Table 2, mostly focused on the *α*4*β*2 form. The exception is *α*2-I279N, which was coexpressed with both *β*4 [21, 245] and *β*2 [246], with broadly similar results.

Until now, no great alterations have been reported in transcription and membrane expression of mutant subunits [21, 245–257]. However, electrophysiological studies show that all the mutations located outside the pore region (M2) increase nAChR function in both homozygous and heterozygous conditions [21, 231, 245, 246]. The overall effect comes down to a widening of the channel “window” current, that is, the crossover of the activation and desensitization curves. This is determined by a combination of higher sensitivity to the agonists and shift of the desensitization curve [231]. Considering the physiology of heteromeric nAChRs in the brain, it is likely that the main result of these changes is a significant potentiation of the steady state nAChR current in the presence of tonic ACh concentrations.

Mutations located in the M2 segment display a more complex behavior, as differences are frequently observed between the properties of the simulated homo- and heterozygotes obtained by expressing human [164, 165, 225, 228, 238, 239, 248–253] and rat hortologue clones [254–257]. Homozygous channels often display a decrease in maximal current, sometimes accompanied by alteration of conductive properties. The strongest effect is observed in *α*4-S248F, which presents a strong decrease in Ca^2+^ permeability and single-channel conductance, consistent with the position of this residue, close to the selectivity filter, and protruding into the conduction pathway [249]. These features had initially suggested a loss-of-function mechanism for ADNFLE, but more detailed studies in heterozygous condition in new [164, 165] as well as old mutations [251–253] indicated that an increase of nAChR function probably underlies the pathogenesis. The simplest hypothesis to explain the discrepancy between the homo- and the heterozygous condition is that, because M2 segments line the channel pore, when multiple homologous M2 amino acid substitutions are simultaneously present, a decrease in conductive properties are much more likely to appear. If the prevalent receptor forms are (*α*4)_2_(*β*2)_3_ and (*α*4)_3_(*β*2)_2_ [36, 37], in homozygous condition 100% of the receptors contain either two or three mutant subunits. In heterozygotes, we can apply elementary combinatorial analysis [258] under the simplifying assumptions of equal expression and random association of the different subunits. In this case, irrespective of whether the mutant subunit is *α*4 or *β*2, 18.75% of the channels would be WT, 18.75% would be mutant (i.e., containing a full complement of mutant subunits), 18.75% would contain one WT and two mutant subunits, and 43.75% would contain one mutant and two WT subunits. On the other hand, the latter percentage would be almost 47% had we assumed that the fifth subunit of the pentamer is more likely (with probability of 0.75) to be *α* than *β*, as it seems actually the case, based on available evidence [37]. This calculation refers to the case where the mutant subunit is *β*2. Hence, in heterozygous condition, between 60 and 70% of the receptors contain only one or no mutant subunit. This percentage could be even higher *in vivo*, where other subunits, such as *α*5 [19], could associate to *α*4*β*2. Regardless of the underlying mechanism, experimental evidence shows that, in heterozygous condition, gating alterations appear, whose functional effects are broadly similar to those produced by the non-M2 mutations (Table 2). 

Moreover, in the rat hortologues of human M2 mutations expressed in *Xenopus* oocytes, the nAChR regulation by extracellular calcium concentration ([Ca^2+^]_*o*_) is often altered [256, 257]. The neuronal nAChRs are progressively potentiated by [Ca^2+^]_*o*_, up to the physiological concentration of 1 to 2 mM [259, 260]. Increasingly higher [Ca^2+^]_*o*_s produce increasing channel block [261]. The ensuing bell-shaped response to [Ca^2+^]_*o*_ depends on Ca^2+^ binding to several metal binding sites, located within the N-terminal domain. These sites contain typical EF-like helix-loop-helix motifs, with conserved terminal glutamate residues, whose neutralization decreases or completely inhibits the regulation by [Ca^2+^]_*o*_ [262]. In the above mutant *α*4*β*2 nAChRs, the potentiating effect of [Ca^2+^]_*o*_ between 0 and 2 mM was found to be strongly reduced [256]. The effect is attributed to altered allosteric activation [257]. Although these effects have been yet not fully studied in the human clones and the evidence in heterozygotes is incomplete, the decreased sensitivity to [Ca^2+^]_*o*_ may once again turn out to amount to increased receptor function [257]. The two proposed mechanisms are as follow: (a) lower nAChR-dependent GABA release, with ensuing circuit dishinibition; (b) increased glutamate release during bouts of synchronous activity in the neocortex [256, 257].

Overall, evidence in expression systems tends to favor an epileptogenic mechanisms caused by potentiation of neocortical heteromeric nAChR function, which would be consistent with the observation that deleting either *α*4 or *β*2 in murine strains does not lead to seizure facilitation or other aspects of the ADNFLE symptoms [263, 264]. However, a note of caution should be added. Considering that nAChRs have complex pre- and postsynaptic roles in the brain and that long-term exposure to agonists modulates the *α*4*β*2 receptor expression [35, 265–267], it would be unsafe to rule out the possibility that similar pathological effects are produced by different mechanisms, especially in families or individuals with different genetic backgrounds.

## 10. Animal Models of ADNFLE

The above studies leave us with the problem of understanding (1) whether the nAChR alterations observed *in vitro* reproduce the physiological situation; (2) how hyperfunctional nAChRs can lead to ADNFLE; (3) whether and in which conditions hypofunctional channels can produce similar effects. Several animal models were recently generated to address these issues. Not surprisingly, no mutant strain perfectly reproduces the human pathology. However, they mimic several features of ANDFLE in that they often induce alterations in arousal and sleep physiology that may be accompanied by spontaneous seizures or decreased threshold to seizure development. Initially, knock-in murine strains were produced which express mutations that, although not found in ADNFLE families, confer to the receptor the hypersensitivity that is often observed The updated reference in ADNFLE mutations. In particular, hypersensitive *α*4*β*2 nAChRs were obtained by using *α*4 subunits carrying amino acid substitutions in the L9' position of M2 (corresponding to L251 of the human clone). Substitution with Ser produces much decreased concentration threshold for nicotine-induced seizures [268, 269], although the overall phenotype is probably too severe to faithfully represent the ADNFLE features, with frequent lethality in homozygous condition. A milder phenotype is observed when L9' is substituted with Ala [270, 271]. These strains do not show spontaneous seizures. However, they present at 5-fold decrease of the nicotine dose necessary to induced seizures and more fragmented NREM sleep. Moreover, the seizures in mutants present features more analogous to those observed in ADNFLE, with hyperkinetic movements and asymmetrical posturing [271].

The first heterozygous ADNFLE murine models were produced on C57BL/6J genetic background expressing either *α*4-S252F or *α*4-+L264, respectively, homologous to the human mutant subunits *α*4-S248F and *α*4-(776ins3) [183]. These mice show recurrent seizures accompanied, at the cellular level, by potentiation of the nicotine-induced GABA release in layer II/III of the prefrontal cortex. Seizures do not seem to be entrained to the sleep-wake cycle. Overall, these strains may represent a severe model of ADNFLE, as human patients present a spectrum of clinical manifestations and only about one-third display clear epileptiform EEG during the crises [220]. Perhaps not surprisingly, the effects of these mutations are strain dependent. A second murine model expressing *α*4-S248F was generated on a mixed CD1-129/Sv background. This strain displayed a milder phenotype, with nicotine-induced dystonic arousal complex similar to the motor features of human ADNFLE, but not spontaneous epileptiform EEG alteration [264]. 

Zhu et al. [272] recently generated transgenic rats expressing *α*4-S284L, corresponding to S252L of the human sequence. These rats showed a spectrum of seizure phenotypes during SWS similar to those observed in humans. This extension of the studied mammalian species is welcome because, although the treatments that lead to epileptiform insurgence in rodent brain slices are similar to those effective in human tissue samples [273], the morphological and neurophysiological differences between rats and mice make these models complementary [273, 274]. From a neurophysiological point of view, mutant rats exhibited attenuation of synaptic and extrasynaptic GABAergic transmission and abnormal glutamate release during SWS. A knock-in model for *β*2-V287L was also recently made available by Xu and colleagues [275]. Once again, mice display a clearly disturbed sleep pattern, with significant increase in activity during the light period (corresponding to the rest period, in mice). Animals also show increased anxiety-related behaviour, but no overt seizure phenotype. No neurophysiological data are yet available on this strain.

In general, the above knock-in models show little overt morphological changes in the brain, in agreement with what is observed in ADNFLE patients. Moreover, the observed phenotypes confirm the notion that the arousal system and the sleep-waking cycle are very sensitive to alterations of heteromeric nAChR-mediated transmission. Nonetheless, the phenotype differences between strains make the implications for epileptogenesis not straightforward. The neurophysiological characterization, incomplete as it may be, indicates that the presence of ADNFLE mutations alters neurotransmitter release in the frontal cortex, especially GABA. This is in line with the known roles of heteromeric nAChRs in the modulation of neurotransmitter release in the frontal cortex and thus encourages deeper physiological studies in these animals, giving perhaps more attention to the thalamocortical interplay, which has been so far scarcely attended to.

As in other epileptic forms, the next generation of murine strains will need to address more specific questions related to the mechanisms of seizures and their developmental origin, by attempting the production of targeted or conditional expression of the mutant subunits [274]. One such attempt has been conducted by Manfredi and colleagues [276], who developed ADNFLE murine strains conditionally expressing the *β*2-V287L subunit, in a doxycycline-dependent way (“TET-off” system; [277]). Expression of *β*2-V287L produces a spontaneous epileptic phenotype, whose penetrance and severity depend on gene dosage. Seizures generally occur during periods of increased delta wave activity, mostly during the light period, which corresponds to the resting-sleeping phase, in mouse. Interestingly, silencing the transgene expression in adult age did not revert the epileptic phenotype [276]. This suggests that the epileptogenic action of *β*2-V287L could take place during brain development. In fact, silencing the mutant gene with doxycycline between embryonic day 1 (E1) and postnatal day 15 (P15) produces a phenotype identical to the WT. When treatment is interrupted, rapid reexpression of the mutant subunit is observed, but no evidence of seizures [276]. Therefore, to induce the epileptic phenotype, *β*2-V287L needs to be expressed during sensitive phases of brain development [see Section 11.2], suggesting that critical stages of synaptic stabilization are implicated in ADNFLE.

## 11. Implication of Mutant nAChRs in the Pathogenesis of ADNFLE

As has been observed earlier, the clinical aspects as well as the molecular and neurophysiological features of ADNFLE present some heterogeneity. It has in fact been recently observed that it is difficult to explain all the aspects of nocturnal frontal lobe epilepsy with an all-encompassing theory [278]. Some general points can however be made. The appearance of crises almost exclusively during NREM sleep and the generally disturbed sleep in murine models of ADNFLE can be probably attributed to excess of the normal arousal effect of the cholinergic system, which normally controls the transition to both waking and REM stages (see Section 5.2). The fact that the hypermotor seizures are often observed in nocturnal frontal lobe epilepsy is a symptom of subcortical dishinibition [222, 278]. This may be reconciled with the notion that the frontal cortex layer V is the main output to subcortical structures and is also the region more ready to develop epileptiform activity. It should be also added that the local GABAergic innervation is particularly dense in layer V [279], which should make this structure particularly sensitive to the frequently observed alteration of GABAergic transmission in murine models of ADNFLE. Another hypothesis about the mechanism by which altered nAChRs can lead to hyperkinetic activity turns on the fact that dopamine release in the striatum is regulated by nAChRs [280]. In fact, in a cohort of ADNFLE patients, reduced mesostriatal dopamine receptor (D1) expression was observed [281].

### 11.1. Specific Epileptogenic Mechanisms in Mature Circuits

What is known about the nAChR function in the neocortex as well as the results obtained with the available rodent strains suggest the following possible causes of hyperexcitability; some of which are not mutually exclusive.

**(i) Postinhibitory Hyperexcitability**

Hyperfunctional nAChRs may cause excessive GABA release. The ensuing hyperpolarization of pyramidal neurons would deinactivate low-threshold *I*_CaT_ and activate *I*_*H*_ currents, thus making cells more sensitive to postinhibitory rebound [6, 183].

**(ii) Local Circuit Dishinibition**

Neurophysiological evidence shows that reciprocal inhibition between fast-spiking interneurons (mainly basket cells) is operant in the rodents' neocortex [282–284]. Hyperfunctional heteromeric nAChRs could thus potentiate GABA release on interneurons, as recent evidence indicates that reciprocal GABAergic transmission between basket cells can be regulated by heteromeric nAChRs [159]. This process would be more probable during SWS, when excitatory thalamocortical neurons tend to be inhibited [101]. In this state, an upsurge of ACh in the neocortex may shift the balance of the basket cells' network towards reciprocal inhibition, thus facilitating seizure development before the parallel ACh release in the thalamus stimulates the thalamocortical drive. This mechanism did not appear to be operant in Klaassen's mice [183], but it could explain Zhu's [272] results.

**(iii) Increased Glutamatergic Drive from Thalamocortical Fibers**

An alternative hypothesis is that, although during stage 2 of NREM sleep the cholinergic tone is generally low, hyperfunctional nAChRs could maintain abnormally high glutamate release, even in the face of low ACh levels. The data obtained in knock-in rats indicate that increased nicotine-dependent glutamate release is indeed present in mutant strains during SWS [272]. However, the possible balance of the effects of mechanisms (ii) and (iii) is difficult to predict, since thalamocortical fibers innervate pyramidal as well as interneurons. Hence, I believe this is a valuable issue for further experimental work, as it could reveal subtle aspects of neocortical transmission.

**(iv) The Contribution of Thalamus: Promotion of Sleep Spindles**

As discussed above, stage 2 of sleep is characterized by sleep spindles. These are generated in the thalamus by the inhibitory action of RT cells onto thalamocortical neurons, but the activity of cortical neurons is essential to synchronize the spindle appearance across wide regions. Sleep spindles are liable to turn into epileptiform activity [50] and could be promoted by nAChR-dependent stimulation of glutamate release onto RT neurons, with ensuing increased release of GABA onto thalamocortical cells [285]. This is another mechanism for which very little experimental work is available.

### 11.2. Possible Developmental Role of nAChRs in Epileptogenesis

Understanding how neuronal circuits alteration during development can facilitate the later establishment of a full-blown epileptic disease is a central problem in epileptology, with many practical implications. Besides leading to better understanding of the natural history of epilepsy, a deeper grasp of this issue will probably lead to much better comprehension of the mechanisms underlying intractable epilepsy [286]. This may depend on stable anatomical or physiological circuit alterations that make the overall network activity difficult to control with the available antiepileptic drugs (AEDs). The results obtained with mice conditionally expressing *β*2-V287L suggest that, in the specific case of ADFNLE, altered nicotinic regulation of brain circuit maturation may contribute to the pathogenesis [276]. Because no evident neuroanatomical alterations are observed in either patients or animal models of ADNFLE [183, 222, 244, 264, 272, 275, 276] the developmental effects must involve subtle changes in the proper synaptic balance that are established during network maturation.

A hallmark of mammalian brain development is the typical “brain growth spurt” occurring around birth. This phase is particularly prolonged in humans, where it begins during the last trimester of pregnancy and continues throughout the first 2 years of life. In mice and rats, the quick brain growth spans the first month of postnatal life, during which extensive neurite extension is followed by establishment of neural connections and synaptogenesis. Myelinization and circuit refinement through cell and synaptic death complete the functional network stabilization [287, 288]. This stage is accompanied by maturation of the cholinergic system and presents an upsurge in nAChR subunit expression, which precedes full maturation of the cortical cholinergic fiber network and expression of muscarinic receptors (reviewed in [288]). In particular, expression of *α*4 and *β*2 subunits reaches a peak towards the end of the second postnatal week [289, 290]. During this period, the density of extrinsic cholinergic innervation [162, 291] increases dramatically in rats. The overall timing seems to be similar in the mouse [159, 163], although data are less extensive. Maturation of the cholinergic system regulates brain maturation, as treatment with various cholinergic ligands (including nicotine) around age P10 produces persistent behavioral and morphological alterations [287]. Moreover, mice that lack *β*2 show region-specific changes in cortical structure [292]. In general, ample evidence that cannot be fully reviewed here shows that the early action of the cholinergic system is important for proper structural and cognitive development [289]. The specific mechanisms by which nAChRs regulate neural circuit wiring in the frontal cortex are only beginning to be elucidated. Importantly, the spontaneous nAChR activity was found to control the switch between the excitatory and inhibitory roles of GABA during development [293]. The progressive substitution of the plasma membrane transporter NKCC1 with KCC2, which is at the basis of this process ([38, 294, 295]), is under control of both homo- and heteromeric nAChR activity [293]. KCC2 appears in different layers at somewhat different stages (after age P3 in layer V pyramidal cells) and its expression is concomitant with the formation of GABAergic synapses [296]. Precise timing of early GABAergic excitation is important for early neuronal development and proper integration of cells into circuits [38, 297]. Therefore, overly active nAChRs in ADNFLE could alter proper timing of this process, with ensuing long-term alteration of neocortical microcircuit architecture and the excitatory to inhibitory balance. 

## 12. Pharmacological Aspects

Control of ADNFLE symptoms is often obtained with carbamazepine [218, 298]. Recent evidence, although still limited, indicates that good results can also be obtained in NFLE patients with topiramate [299] and oxcarbazepine (including in some children refractory to carbamazepine, [300, 301]). Oxcarbazepine is a less toxic analog of carbamazepine, which is rapidly metabolized to 10,11-dihydro-10-hydroxy-carbamazepine (MHD), the clinically effective compound [302–304]. Nonetheless, as is often the case in epilepsy, about 30% of the patients are refractory to pharmacologic treatment [220]. What is worse, in the case of ADNFLE (and NFLE in general), extreme caution must be exerted in attempting surgical therapy, considering the widespread distribution of the mutant receptors in the brain and the current uncertainties about the precise pathogenic mechanisms [222]. Therefore, more thorough pharmacological studies seem particularly urgent for these diseases.

Most AEDs exert their action by targeting ion channels. By far the best characterized effects concern AED modulation of voltage-gated Na^+^ channels [305]. In these, carbamazepine retards the recovery from channel inactivation [306, 307]. Oxcarbazepine and MHD, although less well characterized, are thought to produce similar effects [305, 308]. Topiramate also produces a spectrum of inhibitory actions on voltage-gated Na^+^ channels [309, 310]. However, growing evidence indicates that the action of many common AEDs is far from being specific. Importantly, from our standpoint, several AEDs have been found to modulate ligand-gated channels. In particular, *α*4*β*2 nAChRs are blocked by carbamazepine [311], oxcarbazepine/MHD [245], and lamotrigine [312]. The principal mechanism is probably block of the open channel [311]. The altered pharmacological properties observed in many ADNFLE mutations thus suggested that mutant nAChRs may also respond differently to AEDs. In fact, ADNFLE mutations tend to increase the *α*4*β*2 nAChR sensitivity to AEDs [245, 253, 311], although the opposite effect was observed with *α*2*β*2 [246]. These observations could partly explain the particular efficacy of carbamazepine-related drugs on ADNFLE. Irrespective of their possible clinical significance, which is presently uncertain, these results certainly suggest possible medicinal chemistry strategies aimed at producing new generation compounds with different potency on Na^+^ and ligand-gated channels, for attempting more selective ion channel targeting for therapeutic applications. These compounds could be also tested during brain circuit development, to specifically modulate the relevant ion channels during the sensitive stages of neuronal connection establishment and refinement.

## Figures and Tables

**Table 1:** Ionotropic ACh and GABA receptors. Nomenclature and related monogenic epilepsies.

Receptor	Subunit	Gene	Epilepsy
Muscle nAChR	*α*_1_	CHRNA1	
	*β*_1_	CHRNB1	
	*γ*	CHRNG	
	*δ*	CHRND	
	*ε*	CHRNE	

Neuronal nAChR	*α*_2_	CHRNA2	Sleep-related epilepsy
	*α*_3_	CHRNA3	
	*α*_4_	CHRNA4	ADNFLE
	*α*_5_–*α*_10_	CHRNA5–CHRNA10	
	*β*_2_	CHRNB2	ADNFLE
	*β*_3_	CHRNB3	
	*β*_4_	CHRNB4	

GABA receptors	*α*_1_	GABRA1	JME, CAE
	*α*_2_–*α*_6_	GABRA2–GABRA6	
	*β*_1_	GABRB1	
	*β*_2_	GABRB2	
	*β*_3_	GABRB3	CAE
	*γ*_1_	GABRG1	
	*γ*_2_	GABRG2	FS, CAE, DS, GEFS+
	*γ*_3_	GABRG3	
	*δ*	GABRD	FS, GEFS+
	*ε*	GABRE	
	*θ*	GABRQ	
	*ρ*_1_–*ρ*_3_	GABRR1–GABRR3	
	*π*	GABRP	

Legend. ADNFLE: autosomal dominant nocturnal frontal lobe epilepsy; CAE: childhood absence epilepsy; DS: Dravet syndrome; GEFS+: generalized epilepsy with febrile seizures plus; JME: juvenile myoclonic epilepsy. For reference about GABA receptors and epilepsy, see [48].

**Table 2:** Functional properties of mutant nAChRs linked to sleep-related epilepsy.

Mutant	Clone	Cell type	*γ*	*I*_max_			Potentiation by [Ca^2+^]_*o*_		Sensitivity to ACh		*τ*_DES_	References
				Homo	Het	*P*_Ca_	Homo	Het	Homo	Het
*α*4-S248F	Human	*Xenopus *	nd	nd	nd	nd	nd	nd	no	nd	↓↓	[248, 250, 251]
	Human	*Xenopus *	↓	↓	nd	↓↓	nd	nd	no	nd	↓↓	[249]
	Human	*Xenopus *	nd	↓	no	↓	nd	nd	↑	↑↑	↓↓	[252, 253]
	Rat	*Xenopus *	↓	↓	nd		↓↓	nd	no	nd	↓↓	[254–257]
*α*4-S252L	Human	*Xenopus *	nd	↓	no	no	nd	nd	↑↑	↑↑	↓	[252, 253]
	Rat	*Xenopus *	nd	no	nd	nd	nd	nd	(↑)	nd	↓	[254]
	Rat	*Xenopus *	nd	nd	nd	nd	↓	↓	no	nd	↓	[256, 257]
*α*4-776ins3	Human	*Xenopus *	nd	no	no	↓	↓	nd	↑↑	↑↑	↑	[225, 250, 252, 253]
	Rat	*Xenopus *	(↑)	no	nd	↓	↓	↓	no	nd	no	[254, 256, 257]
*α*4-T265I	Human	*Xenopus *	nd	(↓)	(↓)	nd	nd	nd	↑	↑	no	[228, 253]
*β*2-V287L	Human	HEK	no∗	↓	no	no	↓ ∗	no∗	(↑)	nd	↑↑	[164]
	Rat	*Xenopus *	(↓)	nd	nd	nd	↓	nd	(↑)	nd	no	[256, 257]
*β*2-V287M	Human	*Xenopus *	nd	↓↓	no	nd	nd	nd	↑↑	↑↑	no	[165, 252]
		HEK	no									[231]
	Rat	*Xenopus *	nd	nd	nd	nd	↓	↓	(↑)	nd	no	[256, 257]
*β*2-I312M	Human	*Xenopus *	nd	nd	↑↑	nd	nd		↑↑	↑↑	no	[230]
*β*2-L301V	Human	*Xenopus *	nd	↑↑	↑↑	nd	nd		↑	↑	no	[231]
		HEK/GH4-C1	no								no	
*β*2-V308A	Human	*Xenopus *	nd	↑	↑	nd	nd		↑	↑	no	[231]
		HEK/GH4-C1	no								no	
*α*2-I279N	Human	HEK (with *β*4)	no∗	no	no	nd	nd		↑↑	↑	no	[21, 245]
		*Xenopus *(with *β*2)	nd	nd	nd	nd	nd		↑↑	↑	no	[246]

Legend: *γ*: single-channel conductance; homo: homozygous condition; hetero: heterozygous condition; *I*_max_: current measured in the presence of saturating agonist concentrations; nd: not determined; *P*_Ca_: permeability to Ca^2+^; *τ*_DES_: monoexponential time constant of desensitization; ↑: increase; (↑): small increase; ↑↑: strong increase; ↓: decrease; (↓): small decrease; ↓↓: strong decrease. ∗A. Becchetti and C. Di Resta, unpublished results.

## Abbreviations

ACh: Acetylcholine
AChE: Acetylcholinesterase
ADNFLE: Autosomal dominant nocturnal frontal lobe epilepsy
AED: Antiepileptic drug
CAE: Childhood absence epilepsy
ChAT: Choline acetyltransferase
CNS: Central nervous system
ChoT: Choline transporter
CoA: Coenzyme A
CRH: Corticotrophin-releasing hormone
DS: Dravet syndrome
EC_50_: Half-effective concentration
EEG: Electroencephalography
EPSP: Excitatory postsynaptic potential
FS: Febrile seizures
GABA: *γ*-amino butyric acid
GAD: Glutamic acid decarboxylase
GAT: GABA transporter
GEFS+: Generalized epilepsy with febrile seizures plus
5-HT_3_: 5-Hydroxytryptamine
*I*_CaT_: T-type calcium currents
*I*_H_: Hyperpolarization-activated currents
IPSP: Inhibitory postsynaptic potential
JME: Juvenile myoclonic epilepsy
KCC: K^+^-Cl^−^ cotransporter
mAChR: Muscarinic acetylcholine receptor
MHD: 10,11-Dihydro-10-hydroxy-carbamazepine
nAChR: Neuronal nicotinic acetylcholine receptor
NKCC: Na^+^-K^+^-Cl^−^ cotransporter
NFLE: Nocturnal frontal lobe epilepsy
NREM: Non-REM
REM: Rapid eye movements
RT: Reticular thalamic nucleus
SWS: Slow wave sleep
VAChT: Vesicular acetylcholine transporter
VGAT: Vesicular GABA transporter
VIAAT: Vesicular inhibitory amino acid transporter
WT: Wild type.
